# Nursing students’ knowledge on the management of peripheral venous catheters at Wollega University

**DOI:** 10.1371/journal.pone.0238881

**Published:** 2020-09-17

**Authors:** Werku Etafa, Bizuneh Wakuma, Reta Tsegaye, Tagay Takele

**Affiliations:** 1 School of Nursing and Midwifery, Institute of Health Sciences, Wollega University, Nekemte, Ethiopia; 2 College of Natural and Computational Science, Wollega University, Nekemte, Ethiopia; University Magna Graecia of Catanzaro, ITALY

## Abstract

**Background:**

Adherence to the best standards of nursing practice is the fundamental principle to improve patient outcome and prevent nursing procedure related-infections. A peripheral venous catheter (PVC) is the most common invasive procedure performed in nursing care. Its poor performance could expose patients to bloodstream-related infections. The present study aimed to assess post-basic nursing students’ knowledge of evidence-based guidelines on the management of peripheral venous catheters.

**Methods:**

A cross-sectional study design was conducted on May 01–03, 2019, using a convenient sample 239 among post-basic nursing students in Wollega University.

**Results:**

The study result showed that nursing students’ had a low mean (4.1±1.52) of knowledge about PVC procedure management. Only 41% of the respondents have adhered to recommendations of CDC guidelines. Among the provided options of the items, none achieved 100% correct answers. The majority of nursing students (77%) responded that antiseptic handwashing is always performed before insertion of PVCs. Meanwhile, few students (5%) correctly answered that the infusion set is recommended to be removed after 96 hours when neither lipids nor blood products are administered. In multivariable regression analysis, nursing students who had received training (AOR = 2.9, 95% CI (1.6, 5.1)) and who were younger (AOR = 2.4, 95% CI (1.3, 4.3)) significantly associated with a higher score of knowledge.

**Conclusions:**

This study finding shows that an overall level of knowledge of post-basic nursing students is inadequate. Measurements such as an increase in the provision of adequate training by nurses educators based on evidence-based guidelines could improve the post-basic nursing students’ knowledge.

## Introduction

Venipuncture is the introduction of a needle into a vein to obtain a representative sample of the circulating blood for hematological, biochemical, or bacteriological analysis [1]. Vascular access device (VAD) is equipment that provides access to the patient's vascular system [2]. Peripheral venous catheters (PVC) are the most frequently utilized devices [3]. A peripheral intravenous catheter insertion is the most common invasive hospital procedure performed worldwide [3, 4]. It is used for the administration of medications, intravenous fluids, and blood products [5].

It was reported that more than a billion peripheral intravenous catheters are inserted for hospitalized patients worldwide each year [6]. In the United States, approximately 300 million PVCs are used annually [7]. It is estimated that more than half (59% to 70%) of admitted patients require a peripheral venous line during their hospital stay [3, 4]. However, there is no evidence about the prevalence of PVC utilization among hospitalized patients in Ethiopia.

PVCs are associated with potential complications including hospital-acquired bloodstream infection, thrombophlebitis, extravasation, and pain/discomfort [3–5, 8]. Phlebitis is the most frequent complication of intravenous (IV) infusion that may occur in up to 96% of all patients [9]. Catheter-related bloodstream infections prolong hospitalization by 7 to 14 days, survivors average an additional 24 days in the hospital and an estimated cost of treatment range from $3000 to $56 167 [10].

Nurses are at the forefront of providing IV therapy [11]. It is declared that only trained and competent staff using strict aseptic techniques should be involved in IV or cannula care (RCN, 2005) [12]. Additionally, the Nursing and Midwifery Council (NMC), (2008) assured all practitioners must deliver care based on the best available evidence and/or best practice, and it must be kept up-to-date throughout each health professional’s working life for safe and effective practice [13]. Better knowledge and skill of nurses can minimize infusion-related complications and affect patient safety, satisfaction, health care costs, and length of hospital stay [11].

Several nursing activities are expected to be known by nursing students during their study period [14]. Nursing students’ education should be relevant, has good quality, and adhered to their future professional [15]. A nursing or medical student requires a summed up of theoretical knowledge and skills to effectively serve with their profession [16]. Despite the usefulness of clinical guidelines, it is declared that there is still a lack of knowledge adherence to guidelines [17]. Students have different levels of knowledge [18]. Assessing nursing students’ knowledge about PVC management is essential to recognize their level of understanding about PVC [14]. However, there are limited pieces of evidence on nursing students’ knowledge of evidence-based guidelines on the peripheral venous catheter are limited [18].

The Center for Disease Control and Prevention (CDC) had developed guidelines for the Prevention of Intravascular Catheter-related Infections for healthcare workers [19]. Based on these guidelines recommendations, studies have been conducted to assess nurses’ adherence to these guidelines. A multicenter survey from Italy reported that the low mean level (mean = 5.3) of staff nurses’ knowledge [20]. Similarly, a self-administered survey among Spanish pediatric and neonatal intensive care unit (ICU) health care workers estimated 5.61 of a mean knowledge score [21]. In Australia and New Zealand, a survey of pediatric ICU nurses' knowledge found the mean total knowledge score of 5.5 [22]. Likewise, a pilot study among Jordanian oncology nurses displayed a low mean level of knowledge (2.6) [23]. A survey among nursing students in Italy evaluated a low mean knowledge score (64) [18].

Furthermore, the study in Sweden suggested that nursing students can have adequate knowledge of venipuncture knowledge when assessed by using more than one clinical procedure [14]. Evidence showed that a better test score was associated with a higher level of education, an increased year of training experience, wards attended and an area of work [24, 25]. Training and educating nurses on PVC care had a noticeable improvement in patient safety [26, 27].

To our knowledge, this is the first study aimed to evaluate post-basic nursing students’ knowledge using evidence-based guidelines to prevent infection related to a peripheral venous catheter in Ethiopia. The finding of the present study is a useful baseline for future similar studies for improving nursing students’ knowledge. It also gives direction to Wollega University nursing educators, driving forces, for their future roles to determine nursing education curriculum and standards, and prepare students to successfully acquire knowledge. Because of the impact of student nurses’ knowledge on patient safety and outcome, the current study aimed to examine post-basic nursing students’ knowledge of evidence-based guidelines about peripheral venous catheter procedure management.

## Methods

### Study design and setting

The cross-sectional study design was performed from May 01 to May 03, 2019, at Wollega University main campus found in Nekemte town, which is 330km from the capital city of Ethiopia, Addis Ababa. Wollega University is training nursing students in a Bachelor of Science degree in the nursing program. Nurses who are training after enrolled in a diploma at nursing schools will be admitted to university if they have a certificate of qualification exam and a minimum of two years of full-time clinical nursing experience only. These students will enjoy one of the nursing specialty programs at Wollega University: Pediatrics, Neonatal, Surgical, Psychiatry, Ophthalmic, Operation theatre, and Emergency and Critical Care Nursing. They will stay for five semesters at the university to complete their first-degree training.

### Study population and sample size

All post-basic nursing students who were studying at the time of data collection (n = 311) were included in the study. The sample size (n = 267) was determined by a single population proportion formula using the Cochran formula (d = 0.06, p = 0.05) and 10% non-response rate. Then, we obtained the final sample size (n = 293). Since the total number of post-basic nursing students in Wollega University was 311, the study involved all of them to increase the power of the study. A convenience sample was employed to collect data from all first, second and third-year post-basic nursing students who attended theoretical lessons about the management of PVCs (expected from the first year of the degree program). Nurses who were seriously sick/ill and missed class during the data collection period were excluded from the study.

### Study instrument

A questionnaire used for data collection had two sections. The first section was the participants’ demographic data including previous workplace, age, gender, current program studying, year of study, training received, place of training, and the number of wards practiced. The second section was participants’ knowledge assessing tool adopted from Cicolini et al. [20]. Cicolini et al [20] tested the reliability and validity of the questionnaire, and reported that the items were reliable, valid and had a difficulty index ranges from 0.4–0.97. The instrument was used across different countries by different authors [18–23].

A questionnaire was translated into the local language Afan Oromo to avoid misinterpretation. The validity of the translated questionnaire was confirmed using face and content validity as well as two-experienced nursing researchers and educators. Experts also determined the time required for the participants to complete a questionnaire. Permission to use the questionnaire obtained from the corresponding author [20].

Items used for testing post-basic nursing students’ knowledge consisted of 10 multiple-choice questions with only one possible correct answer, two distractors, and the answering option “I do not know” to equivocate guessing. Each correct answer was given one point, whereas distractors and the “I do not know” option responses were counted as zero; therefore, total scores could range from zero (0) to ten (10). Zero and ten indicate the lowest and highest knowledge score of participants about PVCs related infection management, respectively.

### Data collection procedure

Initially, we took a permission letter from the School of Nursing and Midwifery Ethical Review Committee and distributed it to each department involved in the study. We asked department heads to supervise the study and assign one of their nursing educators for facilitating data. The department heads were responsible to ensure completion of the questionnaire, avoid consulting other students and resources/references needed to answer the items. The participants were informed about the purpose of the study, to individually administer, complete and 20 minutes allowed for returning the questionnaire. Questionnaires were distributed and administered to all voluntary participants available during the data collection at the same. Data facilitators collected each filled questionnaire in an envelope to guarantee the confidentiality and anonymity

### Data analysis

Data obtained were coded and entered into the computer using EPI data version 3.1 statistical packages. The Statistical Package for Social Sciences (SPSS) version 20.0 (IBM Corporation, Armonk, NY) used for data analysis. Descriptive statistics for categorical variables were synthesized as frequencies and percentages whereas for continuous variables they were summarized as mean and standard deviation. Tables, figures and text words were used to describe variable in the study. The mean score attained from the scale was used to measure student nurses’ knowledge of PVC management. A binary logistic regression analysis was used to test the effect of demographics on student nurses’ knowledge of PVC management. The level of significance was set at p-value < 0.05.

### Ethics approval and consent to participate

This study has been reviewed and approved by Wollega University, School of Nursing and Midwifery Research Ethical Review Committee. All volunteer nursing students were invited to take part in the study. They provided both informed and written information about the aim of the study and its procedures. Written informed consent was taken from all participants before enrolled to participate in the study. All voluntary participants were provided it by filling it in the written questionnaire. The participants also were ensured about the anonymity and confidentiality of the data as well as voluntary participation in the study. Furthermore, the students also were informed that they could withdraw from the study at any time they wanted.

## Results

### Demographic characteristics of the participants

The analysis was made based on 239 post-basic nursing students who fully participated in the study, for a response rate of 76.8%. The age of respondents ranges from 22 to 37 years, with a mean age of 28.58 ± 3.19. Male participant nurses were 167 (69.9%). More than half (54.4%) of them had been working in hospitals. Meanwhile, 145(60.7) had participated in the procedures of PVC management training (Table 1).

**Table 1:** Post-basic nursing students’ demographic characteristics, Wollega University, 2019 (N = 239).

Variables	Frequency	Percentage	
Previous workplace			
Hospital	130	54.4	
Health center	86	36.0	
Others	23	9.6	
Age (years) (Mean = 28.58 ± 3.19)			
20–24	16	6.7	
25–29	143	59.9	
30–34	62	25.9	
> = 35	18	7.5	
Sex			
Male	167		69.9
Female	72		30.1
Current study program in nursing			
Pediatrics	54		22.6
Neonatal	32		13.4
Surgical	28		11.7
Psychiatry	24		10.0
Emergency	49		20.5
Operation theatre	29		12.2
Ophthalmic	23		9.6
Year of study			
First-year	111		46.4
Second-year	56		23.4
Third-year	72		30.2
Training received about peripheral venous catheters			
Yes	145		60.7
No	94		39.3
Place of training			
University	13		9.0
College	88		60.7
Workplace	44		30.3
Recent ward/unit served			
Primary health care	40		16.7
Medial	23		9.6
Surgical	23		9.6
Pediatrics	39		16.4
Maternity	26		10.9
Emergency	54		22.6
Others `	34		14.2
Number of wards served (Mean = 2.41± 2.13)			
1–2	55		23.0
3–5	100		41.8
> = 6	15		6.3
None	69		28.9

### Knowledge of respondents on the peripheral venous catheter

Student nurses scored equal or above the mean score had good knowledge otherwise categorized to poor knowledge about PVCs management. The mean, median, and standard deviation scores of knowledge testing items were 4.17, 4 and 1.52, respectively. More than half (n = 141, 59%) of the respondents scored below the mean on PVC knowledge testing items. The percentages of correct answers extended from 5% to 77% relying on the analysis of 10 items. Among the provided options of the items, none achieved 100% correct answers. The highest percentages of correct answers pertained to item number 2 which declared that it is recommended to always perform an antiseptic hand wash before insertion of PVCs” (n = 184, 77%). On the other hand, few students (n = 12, 5%) correctly answered item number 10 which stated the infusion set is recommended to remove after 96 hours when neither lipids nor blood products are administered. Similarly, 23.4% of the participants answered correctly about the importance of chlorhexidine gluconate solution with alcohol to disinfect the catheter insertion site (Table 2).

**Table 2:** Post-basic nursing students’ knowledge about peripheral venous catheter procedure management, Wollega University (N = 239), 2019.

Knowledge test items	Frequency	Percentage
1. It is recommended to replace peripheral venous catheters (PVCs) routinely…		
A. Yes, every 24 h	76	31.8
B.Yes, every 12 h	20	8.4
C. Yes, every 72–96 h *	111	46.4
D. I do not know	32	13.4
2. It is recommended to perform an antiseptic hand wash before insertion of PVCs…		
A. No, it's sufficient to wash hands with a non-antimicrobial soap	32	13.4
B. No, you do this only for invasive procedures	15	6.3
C. Yes, always *	184	77
D. I do not know	8	3.3
3. It is recommended to use an aseptic technique during connecting/disconnecting the infusive lines…		
A. Yes, always *	145	60.7
B. No, it's sufficient to wash hands with an antimicrobial soap	13	5.4
C. No, because it increases the risk of infection	65	27.2
D. I do not know	16	6.7
4. It is recommended to use steel needles (butterfly type) for the administration of drugs…		
A. No, because they might cause tissue necrosis if extravasation occurs *	90	37.7
B. Yes, if I have to inject drugs for a short time	91	38.1
C. Yes, always	40	16.7
D. I do not know	18	7.5
5. It is recommended to change the dressing on the catheter insertion site…		
A. On a daily basis	72	30.1
B. Every 3 days	30	12.6
C. When indicated (soiled, loosened, …) *	11	49
D. I do not know	20	8.4
6. It is recommended to cover up the catheter insertion site with…		
A. Polyurethane dressing (transparent, semipermeable)	65	27.2
B. Gauze dressing	70	29.3
C. Both are recommended because the type of dressing does not affect the risk for catheter-related infections *	80	33.5
D. I do not know	24	10.0
7. It is recommended to disinfect the catheter insertion site with…		
A. >0.5% chlorhexidine gluconate solution with alcohol *	56	23.4
B. 2% chlorhexidine solution with alcohol	52	21.8
C. 10% alcohol	115	48.1
D. I do not know	16	6.7
8. It is recommended to apply an antibiotic ointment at the insertion site of a PVC…		
A. Yes, because it decreases the risk for catheter-related infections	71	29.7
B. No, because it causes antibiotic resistance *	68	28.5
C. No, because it does not decrease the risk for catheter-related infections	59	24.7
D. I do not know	41	17.1
9. When lipid emulsions are administered through a PVC, it is recommended to replace the administration set…		
A. Within 24 h *	135	56.5
B. Every 72 h	49	20.5
C. Every 96 h	8	3.3
D. I do not know	47	19.7
10. When neither lipid emulsions nor blood products are administered through a PVC it is recommended to replace the infusion set		
A. Every 24 h	95	39.8
B. Every 72 h	82	34.3
C. Every 96 h *	12	5.0
D. I do not know	50	20.9

### Predictors of nursing students’ knowledge of peripheral venous catheter procedure management

The binary and multivariable logistic regression methods were performed in the analysis of predictors of the dependent variables. A binary logistic regression analysis was used to assess the associations between the independent and dependent variable. Those variables showed association with dependent variable at p<0.05 in the bivariate analysis were training and age of the participants. The multivariable logistic regression analysis was done associating age and training with the dependent variable (mean of the knowledge scores).

The training was significantly associated with the knowledge of respondents on a peripheral venous catheter. Those nursing students who had training 2.9 times more likely knowledgeable as compared with those who had no training [AOR = 2.9, 95% CI = (1.6, 5.1)]. The age of the respondents was also significantly associated with the knowledge of respondents on peripheral venous catheter procedure management. Those students who were aged 20–29 years were 2.4 times more likely knowledgeable than nurses aged 30–39 years [AOR = 2.4, 95% CI = (1.3, 4.3)] (Table 3).

**Table 3:** Predictors of post-basic nursing students’ knowledge on peripheral venous catheter procedure management, Wollega University, 2019 (N = 239).

Variables	Knowledgeable	Not knowledgeable	COR (95%CI)	AOR (95%CI)	P-value
**Age (years)**					
20–29	73	86	1.86 (1.06–3.29)	2.4 (1.3–4.3)*	0.005
30–39	25	55	1.00	1.00	
**Training**					
Yes	71	74	2.38 (1.37, 4.14)	2.96 (1.6–5.1)*	<0.001
No	27	67	1.00	1.00	

## Discussion

This study evaluated post-basic nursing students’ knowledge of PVCs procedure management. It reflected a low mean total score (4.1±1.52). This report revealed an unsatisfactory level of knowledge of CDC's main recommendations [19]. The present study result is lower than the previously reported results in Italy, Australia and New Zealand (mean = 5.5), and Spain [18, 20–22] However, it is higher than the study reported among Jordan oncology nurses [23]. Lack of refreshment by nursing educators about PVC procedures, weak curricula, and poor attention by nurse educators could reflect the low mean of knowledge score.

Although evidence suggested that high knowledge levels did not necessarily translate into good clinical practice [28], in Ethiopia, we bear that due to lack/absence of resources necessarily initiate nursing students for inadequate knowledge of peripheral venous catheters. These resources could be the lack of experienced and skilled nurse educators who are considered as input for nursing students. Nurse educators in our country (Ethiopia) are hired to university for teaching as graduates from the university with null experience in clinical service, not strictly adhere to nursing curriculum and standards which in turn impedes training qualified professional nursing students. The second is lack or absence of common national guidelines, lack of sufficient equipment, and less opportunity for updated evidence in nursing areas. Furthermore, post nursing students did not read repeatedly to acquire knowledge because their curricula supported them, paying off less energy for scoring a better score. Therefore, nursing educators’ adherence to the participants’ curriculum as per the standards of nursing practice and providing continuous training programs about PVC procedure management as substantial key improvements recommended strongly.

The study result displayed that the highest percentages of correct answer pertained to item number 2 (always performing an antiseptic hand wash before insertion of PVCs (n = 184, 77%), three (aseptic technique during connecting/disconnecting the infusive lines (145, 60.5%), and nine (replacement of administration set used for lipid emulsions (135, 56.5%) were answered by the majority of nurses correctly.

In particular, regarding recommendation always hand wash before insertion of PVCs, our finding is in harmony with the study findings in Italy among nursing students [18] that reported it is known by 66.5% of nursing students. Similarly, the CDC guidelines [19] suggested hand hygiene should be done before and after inserting an intravascular catheter. However, only 60.5% of the participants answered it correctly which is higher than the result revealed among nursing staff (55.2%) and lower than nursing students’ scores in Italy (78.8%) [18, 20]. Repeatedly teaching/reminding nursing students’ hand washing as the first step to prevent nosocomial infection in several nursing procedures could be the reason for answering it correctly.

In this study, more than half (54.6%) of the participants reported that IV sets should be changed within 24hours when lipid emulsions are administered through a PVC. Nevertheless, more errors are reported from student nurses in our area than nurses and nursing students in Italy [18, 20]. The absence of guidelines, exposure and updated information about PVC management could expose them to answer it incorrectly. Informing and giving the post-basic nursing students the sources of the best evidence for obtaining knowledge about PVC management is essential.

A large number (53. 6%) of post-basic nursing students did not know the correct period of peripheral venous catheters replacement. Nonetheless, there is no need to replace peripheral catheters more frequently than every 72–96 hours to reduce the risk of infection and phlebitis in adults according to the latest CDC guidelines [19]. Either post-basic nursing students may not read repeatedly to acquire relevant knowledge or nurse educators may not educate the students about PVC replacement duration in line with the evidence of CDC's guideline. Moreover, only 54.8% of the participants believed that steel needles are used to administer drugs frequently/ infrequently which contradicts with CDC’s guideline main recommendation [19]. This result matched with the study findings undertaken among nursing staff in Italy [20]. Our participants’ understanding of peripheral venous catheters is very low. Nurse educators at Wollega University should focus on the ways and rationales of PVC management.

Data from this study revealed that only 21.8% of post-basic nursing students are aware that >0.5% chlorhexidine solution with alcohol to disinfect catheter insertion sites is in line with the latest CDC guidelines [19]. Nevertheless, about half (48%) of them knew that 10% of alcohol could be used as an antiseptic solution to the catheter insertion site. However, it is strongly recommended and supported to use either 2% chlorhexidine or 70% alcohol besides a mixture of > 0.5% chlorhexidine and alcohol [19]. These ideas suggested that our participants had no understanding of antiseptic solutions concentration that was able to kill microorganisms on the skin surface. Poor nurse educators knowledge of CDC’s guideline [19] main recommendation could result in producing students not adhering to the guideline.

Certainly, the CDC guidelines [19] recommend avoiding antibiotic ointment applications at the insertion site of PVC as it causes antibiotic resistance. In line with this guideline, less than one-third (28.5%) of student nurses similar to other research works [18, 19, 23], knew that applying antibiotic ointment at the insertion site of a PVC as unimportant. The reason behind for post-basic nursing students to practice antibiotic ointment at insertion sites might be their perception that antibiotics support wound healing. This misconception could be avoided by providing training about PVC management.

Furthermore, this study showed that about 3/4th (74%) of nursing students believed that in patients receiving neither lipid emulsions nor blood products administration sets should be replaced within 24 or 72 hours, while this is only needful after 96 hours. Similarly, a multicenter cross-sectional study among Italian nurses also reported 71.3% grant it indispensable every 72–96 hours. Nevertheless, Cicolini et al. [20] reported 54.6% of the participants concluded it is needful to change it every 24 or 72 hours. Lack of updated information and specific guidelines about peripheral venous catheter management for post-basic nursing students may be the possible reasons. Therefore, the provision of updated evidence-based information and assessing students with early feedback by nurse educators at Wollega University will equip the students with commonly practiced nursing procedures.

The study also highlighted the relationship between student nurses’ demographic characteristics and knowledge of evidence-based guidelines on the management of PVCs. Nevertheless, the training and age of the respondents were significant variables associated with student nurses’ knowledge scores. Nursing students who had received training about PVC procedure were 2.38 times more likely knowledgeable than those who didn’t receive any training about PVC procedure management (AOR = (2.9, CI (1.6, 5.1)). This result is similar to the study reported in Italy. [18], that showed nurses who received training had higher knowledge scores than those who did not receive the training (p < 0.001). Another study also suggested that educational courses and real-time feedback would improve nurses’ performance on PVC procedure [26]. Despite peripheral venous catheter insertion is the most commonly practiced nursing procedure in Ethiopia from our experience, there is deficit attention about PVC procedure management. This might be due to the lack of standardized updated manual and sparse recognition of the wrongdoing of PVCs procedures. Similarly, younger students had higher knowledge scores than older nursing students (AOR = 2.4(1.3, 4.3)). This could be due to lack of a refreshment course or training and absence of updated guidelines or information about the management of peripheral venous catheter procedures.

Moreover, similar to other studies [18, 21, 22] there was no statistically significant relationship/ difference in score between genders. However, two studies [18, 20] reported that a higher knowledge score related to gender, the year of education and the number of wards attended in contrast to our result.

## Conclusions

In general, the study referred to a low mean total of knowledge score of post-basic nursing students that needs to be improved. This resulted from poor adherence to the protocols of clinical practice guidelines that could consequent in poor patient safety and satisfaction.

We found a significant positive association between post-basic nursing students’ knowledge scores and training experience/younger nurses. Nurse educators are vital for maintaining post basic nursing students’ knowledge based on the best pieces of evidence available. Also, the provision of ongoing training, mentoring, and assessing students regularly during theoretical lessons and at the practical site will improve. Further study will be necessary to extend the knowledge of nursing students about procedures of PVCs management involving clinical staff nurses and nurse educators.

### Limitations of the study

The study was monocentric and analyzed from a small size, which may affect the generalization of these findings. However, the cross-sectional study design allows determining associations but not causal relationships in the analysis of potential predictors of knowledge. Besides, a self-report questionnaire can result in a low response rate (under-reporting). However, the strength of this study is using standardized tools of scales to assess nurses’ knowledge about the peripheral venous catheter.

## Supporting information

S1 Data (SAV)

## Abbreviations

PVC: Peripheral Venous Catheters
IV: Intravenous
CDC: Center for Disease Control and Prevention
RCN: Royal Council of Nursing
NMC: Nursing and Midwifery Council
